# Disruption of a Structurally Important Extracellular Element in the Glycine Receptor Leads to Decreased Synaptic Integration and Signaling Resulting in Severe Startle Disease

**DOI:** 10.1523/JNEUROSCI.0009-17.2017

**Published:** 2017-08-16

**Authors:** Natascha Schaefer, Alexandra Berger, Johannes van Brederode, Fang Zheng, Yan Zhang, Sophie Leacock, Laura Littau, Sibylle Jablonka, Sony Malhotra, Maya Topf, Friederike Winter, Daria Davydova, Joseph W. Lynch, Christopher J. Paige, Christian Alzheimer, Robert J. Harvey, Carmen Villmann

**Affiliations:** ^1^Institute of Clinical Neurobiology, Julius-Maximilians-University of Würzburg, 97078 Würzburg, Germany,; ^2^Princess Margaret Cancer Centre, University Health Network, University of Toronto, Toronto, Ontario M5G 2M9, Canada,; ^3^Institute of Physiology and Pathophysiology, and; ^4^Institute of Biochemistry, Friedrich-Alexander-University Erlangen-Nürnberg, 91054 Erlangen-Nürnberg, Germany,; ^5^Queensland Brain Institute, University of Queensland, Brisbane 4072, Australia,; ^6^Department of Pharmacology, UCL School of Pharmacy, London WC1N 1AX, United Kingdom,; ^7^Department of Biochemistry, University of Cambridge, Cambridge CB2 1QW, United Kingdom, and; ^8^Institute of Structural and Molecular Biology, UCL Birkbeck College, London WC1E 7HX, United Kingdom

**Keywords:** β8–β9 loop, fast decay, glycine receptor, hydrogen bond network, shaky, startle disease

## Abstract

Functional impairments or trafficking defects of inhibitory glycine receptors (GlyRs) have been linked to human hyperekplexia/startle disease and autism spectrum disorders. We found that a lack of synaptic integration of GlyRs, together with disrupted receptor function, is responsible for a lethal startle phenotype in a novel spontaneous mouse mutant *shaky*, caused by a missense mutation, Q177K, located in the extracellular β8–β9 loop of the GlyR α1 subunit. Recently, structural data provided evidence that the flexibility of the β8–β9 loop is crucial for conformational transitions during opening and closing of the ion channel and represents a novel allosteric binding site in Cys-loop receptors. We identified the underlying neuropathological mechanisms in male and female *shaky* mice through a combination of protein biochemistry, immunocytochemistry, and both *in vivo* and in *vitro* electrophysiology. Increased expression of the mutant GlyR α1^Q177K^ subunit *in vivo* was not sufficient to compensate for a decrease in synaptic integration of α1^Q177K^β GlyRs. The remaining synaptic heteromeric α1^Q177K^β GlyRs had decreased current amplitudes with significantly faster decay times. This functional disruption reveals an important role for the GlyR α1 subunit β8–β9 loop in initiating rearrangements within the extracellular–transmembrane GlyR interface and that this structural element is vital for inhibitory GlyR function, signaling, and synaptic clustering.

**SIGNIFICANCE STATEMENT** GlyR dysfunction underlies neuromotor deficits in startle disease and autism spectrum disorders. We describe an extracellular GlyR α1 subunit mutation (Q177K) in a novel mouse startle disease mutant *shaky*. Structural data suggest that during signal transduction, large transitions of the β8–β9 loop occur in response to neurotransmitter binding. Disruption of the β8–β9 loop by the Q177K mutation results in a disruption of hydrogen bonds between Q177 and the ligand-binding residue R65. Functionally, the Q177K change resulted in decreased current amplitudes, altered desensitization decay time constants, and reduced GlyR clustering and synaptic strength. The GlyR β8–β9 loop is therefore an essential regulator of conformational rearrangements during ion channel opening and closing.

## Introduction

Glycine receptors (GlyRs) are members of the superfamily of Cys-loop receptors (CLRs), whose structures have recently been resolved by x-ray crystallography or cryo-electron microscopy (EM; Du et al., 2015; Huang et al., 2015). Adult inhibitory GlyRs form pentameric ion channels with a 2α:3β stoichiometry (Grudzinska et al., 2005). Disturbances in glycinergic inhibition are associated with rare disorders such as startle disease (OMIM 149400, hyperekplexia, stiff baby syndrome) and autism spectrum disorders (Harvey et al., 2008; Bode and Lynch, 2014; Pilorge et al., 2016). The genetic causes for startle disease have been defined, with the most common gene *GLRA1* (encoding the GlyR α1 subunit) followed by *SLC6A5* (glycine transporter 2) and *GLRB* (GlyR β subunit; Harvey et al., 2008; Chung et al., 2010; James et al., 2013). Affected patients show exaggerated startle responses following unexpected acoustic or tactile stimuli, stiffness in infancy, tremor, and loss of postural control during startle episodes (Schaefer et al., 2013). GlyRs are important in the spinal cord for feedback control mechanisms in the nerve–muscle circuit to balance motoneuron firing and in the brainstem for the generation of the respiratory rhythm (Bongianni et al., 2010; Schaefer et al., 2012). The current view of startle disease pathology differentiates between functional impairments and biogenesis defects (Bode and Lynch, 2014). A recent study (Schaefer et al., 2015) demonstrated that startle disease mutations also affect GlyR folding and ER processing, suggesting a higher molecular complexity of disease mechanisms than was previously assumed.

Due to a similar startle phenotype in mice carrying *Glra1* (*spasmodic*, *oscillator*) or *Glrb* (*spastic*) mutations, mouse models serve as excellent tools to study the underlying pathological mechanisms. *Oscillator* is a functional GlyR α1 subunit null mutation, while *spasmodic* harbors an A52S missense mutation in the GlyR α1 subunit (β1–β2 loop), leading to decreased ligand affinities but normal life span (Schaefer et al., 2012). The novel spontaneous mouse mutant *shaky* carries a missense mutation in exon 6 of *Glra1*, resulting in a Q177K substitution in the β8–β9 loop of the GlyR α1 subunit extracellular domain (ECD). In contrast to *spasmodic*, homozygous *shaky* mice suffer from serious neuromotor deficits progressively increasing from postnatal day 14 (P14) until death, indicating that disruption of the β8–β9 loop substantially impairs glycinergic function and is incompatible with life.

To date, the β8–β9 loop has mainly been investigated by *in vitro* mutagenesis studies in other CLRs (Thompson et al., 2006; Hibbs et al., 2009). These studies revealed effects on ligand efficacies or affinities, arguing for an involvement of the β8–β9 loop in the ligand-binding process. This view was recently expanded by the identification of the β8–β9 loop forming an allosteric binding site for the antipsychotic compound chlorpromazine in the closely related *Erwinia* ligand-gated ion channel ELIC (Nys et al., 2016). Structural data have demonstrated that the β8–β9 loop is part of the signal transduction unit, transferring the closed state upon ligand-binding into the open configuration and back to the closed ion channel state (Du et al., 2015; Morales-Perez et al., 2016). However, the role of the β8–β9 loop in disease mechanisms is unclear.

Here, we found that the β8–β9 loop is involved in GlyR synaptic clustering as well as neurotransmitter sensitivity and that a defect in these mechanisms causes severe startle disease. In neuronal cultures and spinal cord tissue from *shaky* mice, we observed an increased expression level of GlyR α1^Q177K^, which was an unsuccessful attempt at compensation for an observed lack of GlyR integration into synapses. Decreased agonist efficacy and faster decay times of α1^Q177K^β GlyRs were recorded in artificial synapses and *ex vivo* brainstem slices. Recordings after the onset of neuromotor symptoms revealed significant reductions in current amplitudes, frequencies, and decay times but no changes in rise times in *shaky* homozygotes. Thus, *shaky* represents the first *in vivo* model highlighting that β8–β9 loop is a key regulator of GlyR signaling as it is essential for conformational rearrangements governing both receptor clustering and ion channel gating.

## Materials and Methods

### 

#### 

##### Mouse lines.

The novel mutant mouse strain *shaky* arose as a spontaneous mutation in the animal colony of C. Paige (University Health Network Research, Toronto, ON, Canada) in a mixed 129X1/SvJ/C57BL/6 strain. Mice were transferred into the animal facility of the Institute of Clinical Neurobiology (Würzburg, Germany), where mice were housed under pathogen-free conditions; water and food were available *ad libitum*. All mice were the offspring of heterozygous +/*sh* matings or crosses with 129X1/SvJ wild-type mice. Experiments were approved by the local veterinary authority (Veterinäramt der Stadt Würzburg) and the Ethics Committee of Animal Experiments (i.e., Regierung von Unterfranken, Würzburg, Germany; license #55.2-2531.01-09/14). *Oscillator* and *spasmodic* mice were a gift from C.-M. Becker (Institute of Biochemistry, Friedrich-Alexander University Erlangen-Nürnberg, Erlangen-Nürnberg, Germany) and derived via embryo transfer into our animal facility. For all experiments, animals of both sexes were used.

##### Experimental design and statistical analysis.

For each experiment, the genotype and the sample size are described in the legend of the corresponding figure. Animals of both sexes were used since no significant differences were detected on the basis of sex, so data were therefore pooled. Replicates were performed with tissue from different animals or transfection material from independent transfection rounds, and *n* values are always provided in figure legends. Recordings of transfected cells have been made from at least three independent experiments (except EC_50_ values were recorded from one set of experiments). The number of cells recorded are documented in figure legends. Recordings from slices were made from at least three independent animals, and the numbers of cells recorded from how many animals differed are shown in figure legends. All other experiments have been exhibited at least three times. Statistical significance was calculated using the one-way ANOVA and the unpaired *t* test. The analyses were performed using Origin 6.0 (Microcal Software), ImageJ version 1.50e software (https://imagej.nih.gov/ij/), and Graphics Prism Program version 5.0 (GraphPad Software). All *p* values are given in the Results section.

##### Behavioral analysis.

The neuromotor phenotype of homozygous *Glra1^sh/sh^* mice was investigated by overall visual examination, hindfeet clasping, righting ability, assessment of gait by footprint recordings, body weight, and survival. Videos were recorded with the multi-conditioning system (256060 series) from TSE. Pictures were taken on P14 and P21, and body weight was checked over a period of 6 weeks every 2–3 d.

##### Rotarod.

Mice were tested on a motorized rotarod (grooved rod Ø 4 cm; 11-cm-wide compartments; acceleration speed, 2–20 rpm). The duration of time that mice remained on the rod during rotation was measured with a maximum of 300 s per animal. Diazepam was injected intraperitoneally at a concentration of 0.5 mg/kg in a total volume of 100 μl of sterile PBS. Control animals were injected with PBS.

##### RNA isolation and reverse transcription-PCR.

RNA extraction from tissue was performed as recommended by the manufacturer (PEQLAB) using peqGOLD RNAPure. One microgram of RNA was used for reverse transcription (RT)-PCR. We used RevertAid M-MuLV (Moloney murine leukemia virus) RT (200 U/μl) provided with 5× reaction buffer and deoxy (d) ATP, dCTP, dGTP, dTTP (10 mm each), and random hexamers (50–200 ng; Thermo Fisher Scientific). Two microliters of cDNA were used for amplification of the housekeeping gene β-actin; GlyR α1, α2, and β; gephyrin, and GlyT1 (95°C for 5 min; 25 cycles at 95°C for 30 s, 55°C for 30 s, 72°C for 30 s, and 72°C for 10 min).

##### Cell lines and primary neurons.

Human embryonic kidney 293 (HEK293) cells were grown in minimum essential medium (Thermo Fisher Scientific), supplemented with 10% fetal calf serum, l-glutamine (200 mm), and 50 U/ml penicillin and streptomycin at 37°C and 5% CO_2_. Cells were transiently transfected using a modified calcium phosphate precipitation method. All experiments concerning protein biochemistry with HEK293 cells were performed 48 h after transfection. Primary spinal cord neuronal cultures were prepared at embryonic day 13 (E13). A piece of tail tissue of each embryo was used for genotyping. Briefly, every spinal cord was trypsinized using 1 ml of trypsin/EDTA (1 mg/ml) and 10 μl of DNase I (final concentration, 0.1 mg/ml), incubating the suspension at 37°C for 30 min. Trypsinization was stopped with 100 μl of fetal calf serum (final concentration, 10%). After a three-step trituration, the cells were centrifuged at 300 rpm for 15 min. Trituration was repeated. Cells were plated on a 3 cm dish with four poly-lysine-covered coverslips and incubated at 37°C with 5% CO_2_ at 95% humidity. Spinal cord neurons were grown in neurobasal medium plus 5 ml of l-glutamine (200 mm) plus B27 supplement (Thermo Fisher Scientific) with an exchange of 50% medium after 6 d in culture. Neurons older than day *in vitro* 21 were used for experiments.

##### Membrane preparations and biotinylation of cell surface proteins.

For membrane protein analysis, crude cell membranes were prepared from transfected cells or mouse tissue (Sontheimer et al., 1989). Biotinylation experiments were performed as described previously (Atak et al., 2015).

##### Radioligand-binding assays.

[^3^H]strychnine displacement assays were performed using filtration assays with triplicates of 80 μg of membrane protein. Samples were incubated for 30 min either with 30 mm glycine or buffer B (25 mm potassium phosphate buffer, 200 mm KCl). Then either glycine or buffer B was replaced by a range of [^3^H]strychnine concentrations (1, 10, 20, 50, 100, and 200 nm; specific activity, 30 Ci/mmol; DuPont NEN; Kling et al., 1997). Binding data were analyzed by a nonlinear algorithm provided by the program Origin version 6.0 (Microcal Software).

##### Western blot and immunostaining.

For SDS-PAGE, 11% polyacrylamide gels were freshly prepared, followed by Western blot on nitrocellulose membranes (GE Healthcare). Membranes were blocked for 1 h with 5% BSA in TBS-T (TBS with 1% Tween 20). Primary antibodies were incubated overnight at 4°C. GlyR proteins were detected with the pan-α antibody for GlyRs (mAb4a; 1:500; catalog #146 011, Synaptic Systems), GlyR α1 antibody (mAb2b; 1:1500; catalog #146 003, Synaptic Systems), gephyrin antibody (1:500; catalog #147 003, Synaptic Systems), β-actin (1:5000; catalog #GTX26276, GeneTex), or pan-cadherin (1:1500; catalog #4068, Cell Signaling Technology) served as a loading control. Signals were detected using the ECL Plus System (GE Healthcare). Image quantification for Western blots was performed using ImageJ version 1.50e software (https://imagej.nih.gov/ij/). Data were analyzed using Student's *t* test (ANOVA) or one-way ANOVA, and values below *p < 0.05 were considered significant (**p < 0.01, ***p < 0.001). The values are displayed as the mean ± SEM or as otherwise noted. The graphs were generated using Origin version 6.0 software (Microcal Software).

##### Immunohistochemistry.

Spinal cords were dissected, rapidly frozen on dry ice, and embedded in Tissue-Tek (Sakura Finetek). Transverse slices with a thickness of 8–9 μm were cut with a cryostat (Jung Frigocut 2800E, Leica). Four to six slices were transferred to glass slides (25 × 75 × 1.0 mm; SuperFrostR Plus, Menzel Gläser/Thermo Scientific) and stored for further analysis at −80°C. For immunostaining, tissue slides were fixed for 30 s in ice-cold 2% PFA and immersed once in 50 mm NH_4_Cl following quenching for 30 min in 0.1 mm glycine in PBS. Tissue slices were blocked by 10% normal horse serum in PBS for 1 h at room temperature. The slides were incubated with antibodies GlyR α1 (1:250; mAb2b; catalog #146 111, Synaptic Systems), VGAT (1:300; catalog #131 003, Synaptic Systems), gephyrin (1:200; catalog #147 003, Synaptic Systems), and synapsin (1:300; catalog #574778, Calbiochem) in 10% normal horse serum in PBS overnight at 4°C. After three washing steps with PBS for 10 min each, tissue was incubated with secondary antibodies coupled to Cy3 and Alexa Fluor 488 (1:1000, Dianova) diluted in 10% normal horse serum in PBS for 45 min at 22°C. For staining of the cell nuclei, slides were incubated in Molecular Probes DAPI solution (Thermo Fisher Scientific) diluted 1:1500 in PBS for 10 min at room temperature in a dark chamber. Finally, the slides were mounted with aqua polymount (Polysciences).

##### Confocal microscopy, and image acquisition and analysis.

Images were acquired using an inverted IX81 microscope equipped with an Olympus FV1000 confocal laser scanning system, an FVD10 SPD spectral detector, and diode lasers of 405 nm (DAPI), 495 nm (Alexa Fluor 488), and 550 nm (Cy3). All images shown were acquired with an Olympus UPLSAPO 60× (oil objective; numerical aperture, 1.35) objective. The images were further developed and organized by Adobe Photoshop CS5 and Illustrator software or Corel Photopaint, Corel Graphic Suite ×6.

##### Image analysis for quantification.

Single coverslips were acquired using settings of a photomultiplier identically applied to all samples quantified in one experiment. Maximal projection images were created from confocal stacks using NIH ImageJ 1.50e software (https://imagej.nih.gov/ij/). Nonspecific background was removed using threshold subtraction. In all experiments, clusters of GlyRs were defined semiautomatically by setting rectangular regions of interest (ROIs) with dimensions of approximately 8 × 8 pixels around local intensity maxima in the channel with GlyR α1-specific (mAb2b) staining using OpenView software (Tsuriel et al., 2006). Mean immunofluorescence (IF) intensities were measured in a 4 × 4 pixel box within every ROI in all corresponding channels. Obtained IF intensities were normalized to the mean intensity of control (*shaky* vs wild type). All results of IF analysis are shown as the mean ± SEM. All statistical analyses were performed with GraphPad Prism version 5.0 software (GraphPad Software)

##### Counting of motoneurons.

Mice were deeply anesthetized and transcardially perfused as described previously (Jablonka et al., 2000). Slices were stained with cresyl violet, and motoneurons were counted in every 10th section of the lumbar spinal cord (L1–L6). The raw counts were corrected for double counting of split nucleoli as described previously (Masu et al., 1993). Differences between groups were evaluated with Student's *t* test (unpaired; significance level, *p < 0.05), applying the Graphics Prism Program version 5.0 (GraphPad Software).

##### Molecular modeling of the GlyR α1 subunit Q177K mutation.

The cryo-EM structure [Electron Microscopy Data Bank (EMDB), ID EMD-6345; Protein Data Bank (PDB), ID 3JAE] of the zebrafish GlyR α1 subunit was used to study the structural and functional effects of the Q177K substitution. The position of glycine in the binding site was modeled based on the position of glutamate in the binding site of GluCl (PDB ID, 3RHW) using a superposition of the two receptors with MODELLER (Sali and Blundell, 1993). Flexible fitting was performed using MODELLER/Flex-EM (Topf et al., 2008; Joseph et al., 2016). The nonsynonymous substitution Q177K was modeled into the GlyR using the *swapaa* command in Chimera (Pettersen et al., 2004) based on the Dunbrack backbone-dependent rotamer library (Dunbrack, 2002) and taking into account the lowest clash score, highest number of H-bonds, and highest rotamer probability. Flexible fitting resulted in moving R65 into the EM density.

##### Electrophysiological recordings from transfected cells.

Maximal current amplitudes (*I*_max_) were measured by patch-clamp recordings in a whole-cell configuration from transfected HEK293 cells. Current signals were amplified with an EPC-9 amplifier (HEKA). Whole-cell recordings were performed by the application of ligand using a U-tube system bathing the suspended cell in a laminar flow of solution, giving a time resolution for equilibration of 10–30 ms. Glycine was used at concentrations between 0.3 μm and 3 mm. The external buffer consisted of the following (in mm): 137 NaCl, 5.4 KCl, 1.8 CaCl_2_, 1 MgCl_2_, and 5 HEPES, pH adjusted to 7.4 with NaOH. The internal buffer consisted of the following (in mm): 120 CsCl, 20 N(Et)_4_Cl, 1 CaCl_2_, 2 MgCl_2_, 11 EGTA, and 10 HEPES, pH adjusted to 7.2 with CsOH. Recording pipettes were fabricated from borosilicate capillaries with an open resistance of 4–6 MΩ. Current responses were measured at a holding potential of −60 mV. All experiments were performed at 22°C. For desensitization analysis, whole-cell current traces were transferred to Microcal Origin version 6.0 (Microcal Software), and the decaying current phase was analyzed using a single exponential function plus a constant, as shown in Equation 1:

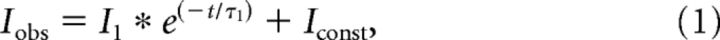
 where *I*_obs_ is the observed total current amplitude, *I*_1_ is the fraction of current desensitizing with time constant τ_1_, and *I*_const_ is the amplitude of the nondesensitizing current fraction. A single exponential decay plus a constant term were sufficient to describe desensitization behavior. Functional constants of the coexpressed subunits were compared using a *t* test. A probability of error of *p < 0.05 was considered significant (**p < 0.01, ***p < 0.001).

##### Electrophysiological recordings from artificial synapses.

Experiments were performed as described by Zhang et al. (2015).

##### Brainstem slice preparation and whole-cell recordings.

Electrophysiological experiments were performed on brainstem slices from 18- to 24-d-old mice. After anesthesia and decapitation, brainstem tissue was rapidly removed and immersed in ice-cold “high-sucrose” artificial CSF (aCSF) containing the following (in mm): 75 sucrose, 125 NaCl, 3 KCl, 0.3 CaCl_2_, 7 MgCl_2_, 1.25 NaH_2_PO_4_, 25 NaHCO_3_, and 30 d-glucose and bubbled with carbogen (95% O_2_/5% CO_2_) at pH 7.4. Transverse slices, 250–350 μm thick, containing the PreBötzinger complex (PreBötC) were cut and transferred into warm (35°C) aCSF for 15 min and kept at room temperature thereafter in normal aCSF for at least 1 h before using. Recordings were performed in normal aCSF buffer, pH 7.4, that contained the following (in mm): 125 NaCl, 3 KCl, 2 CaCl_2_, 2 MgCl_2_, 1.25 NaH_2_PO_4_, 25 NaHCO_3_, and 10 d-glucose at 30°C. The brainstem region of interest was identified based on their location relative to nearby landmarks such as the inferior olive, hypoglossal nerve and nucleus ambiguus (for PreBötC). Whole-cell recordings were performed with patch pipettes filled with an internal solution composed of (in mm): 130 CsCl, 3 MgCl_2_, 5 EGTA, 5 HEPES, 2 Na_2_-ATP, 0.3 Na_3_-GTP, and 5 QX-314, pH 7.3. The electrode resistance ranged from 3 to 5 MΩ when filled with internal solution. Whole-cell currents were recorded at a holding potential of −70 mV (corrected for liquid junction potential), filtered (2–6 kHz), and sampled at 20 kHz using a Multiclamp 700B Amplifier in conjunction with a Digidata 1440A interface and pClamp10 software (Molecular Devices).

Constant current pulses (width, 0.01 ms) of 100–400 μA were delivered every 10 s to a bipolar tungsten electrode located in close vicinity to the preBötC to evoke synaptic responses. Glycinergic IPSCs were pharmacologically isolated by ionotropic glutamate receptor antagonist kynurenic acid (KA; 2 mm) and GABA_A_ receptor antagonist bicuculline methiodide (BMI; 10 μm). Glycine (50 μm) was used to induce postsynaptic response in the presence of tetrodotoxin (TTX; 1 μm) and kynurenic acid/bicuculline. Strychnine (10 μm) was applied in some experiments to verify the glycinergic origin of IPSC or to block glycine-induced currents. The input–output relationship of evoked IPSC was plotted with the negative peaks of synaptic response against the stimulating intensity.

Miniature glycinergic IPSCs (mIPSCs) were pharmacologically isolated by perfusing the slices with aCSF in the presence of KA, BMI, and TTX. Individual events were detected with Clampfit software (Molecular Devices) using a template method with amplitude threshold set to 5–6 * σ_noise_. The peak amplitude, 10–90% rise time, 90–10% decay time, and area were measured and averaged over a minimum of 20 events. For mIPSC kinetics, only nonoverlapping events with relatively fast rise times (<2 ms) and a smooth decay were included in the analysis.

## Results

### Impairment of GlyR α1 subunit β8–β9 loop underlies a neuromotor phenotype and lethality in the mouse mutant *shaky*

Our current view on startle disease has focused on GlyR variants that affect either receptor function or biogenesis. However, potential *in vivo* compensatory mechanisms are still a matter of debate. Here, we used a novel mouse startle disease model, *shaky*, to demonstrate that the extracellular GlyR α1 subunit β8–β9 loop is a key structural element for GlyR function, signaling, and receptor clustering. The combined functional disruption and synaptic clustering defects in this model allowed us to study the compensatory mechanisms of both effects *in vivo*.

**Figure 1.**
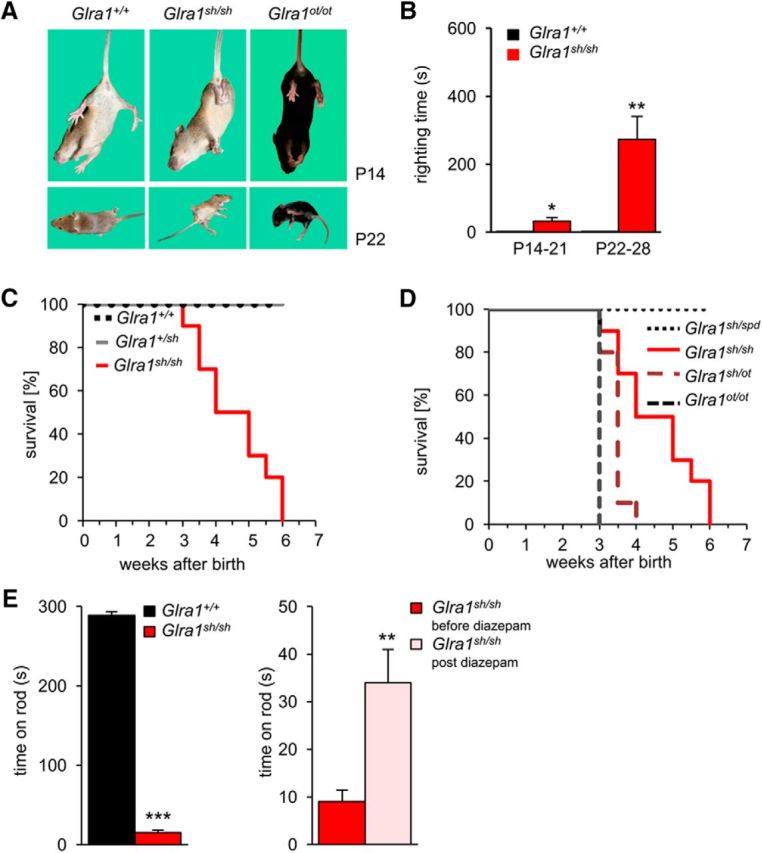
*Shaky* mice display a neuromotor phenotype and carry a *Glra1* mutation. ***A***, Comparison of hindfeet and hindlimb clenching behavior of wild-type *Glra1*^+/+^, *Glra1^sh/sh^*, and *Glra1^ot/ot^* mice at P14 when mice were lifted by the tails, and progressive motor phenotype at P22 (bottom panels) with rigidity in forelimbs, hindlimbs, and tails. ***B***, Impaired righting time of mutant *Glra1^sh/sh^* mice. Time *Glra1^sh/sh^* mice are required to get up at P14 to P21 compared with wild type, prolonged righting time at stage P22 to P28. Cutoff time was 10 min, and 2 of 10 mice older than 21 d timed out. *Glra1*^+/+^, *n* = 7; *Glra1^sh/sh^*, *n* = 10. ***C***, Survival curves of *Glra1^sh/sh^*, *n* = 9; *Glra1*^+/*sh*^, *n* = 19; *Glra1*^+/+^, *n* = 4 mice. *Glra1^sh/sh^* mice die between weeks 3 and 6 of life. ***D***, Survival curves of backcross experiments with GlyR mutant mouse lines *spasmodic* (*spd*) and *oscillator* (*ot*): note that heterozygous *Glra1^sh/ot^* mice die 4 weeks after birth similar to *Glra1^ot/ot^* mice and in contrast to *Glra1*^+/*sh*^ mice. *Glra1^sh/spd^*, *n* = 10; *Glra1^sh/sh^*, *n* = 9; *Glra1^sh/ot^*, *n* = 10; *Glra1^ot/ot^*, *n* = 10. ***E***, Rotarod performance. Wild-type mice had no difficulties staying on the rotating rod for a maximum time of 300 s. *Glra1^sh/sh^* mice were able to remain on the rod for only a few seconds. *Glra1*^+/+^, *n* = 17; *Glra1^sh/sh^*, *n* = 12. *p < 0.05; **p < 0.01; ***p < 0.001.

The spontaneous mouse mutant *shaky* became conspicuous when ∼25% of pups at the age of 2 weeks developed a severe motor defect characterized by typical hindfeet clasping when picked up by their tails (Fig. 1*A*), including tremor, muscle spasms, a twitchy tail, and stiffness. These neuromotor symptoms are similar to *oscillator* mice (Buckwalter et al., 1994), a mouse model of startle disease with a progressive severe phenotype due to truncated nonfunctional GlyR α1 subunit (Fig. 1*A*). During episodes of tremor, *shaky* mice display a hunched, stiff posture (Fig. 1*A*, bottom). The righting ability is poor with a ninefold increase in righting time between weeks 3 and 4 of life [*Glra1*^+/+^, 0.1 ± 0.1 s; *Glra1^sh/sh^*, 32 ± 10 s (*p = 0.0351 at P14–P21); *Glra1*^+/+^, 0.1 ± 0.1 s; *Glra1^sh/sh^* 273 ± 68 s (**p = 0.00079 at P22–P28, *t* test); Fig. 1*B*]. Homozygous mutant mice are usually smaller than their littermates and die at 3–6 weeks of age (Fig. 1*C*). Sequencing of *Glra1* in *shaky* mice (*Glra1^sh/sh^*) and littermate controls revealed two sequence variants: c.T198C in exon 3 (synonymous, p.N38N) and c.C613A in exon 6 (missense, p.Q177K; numbers refer to mature proteins). The identification of a GlyR missense mutation provided a plausible explanation for the observed severe motor phenotype similar to other startle disease mouse models. The synonymous change in exon 3 was not causative and is a common variant that comes from the hybrid C57BL/6129SvJ background (rs26948271) of *shaky*. However, breeding of heterozygotes for the missense mutation Q177K resulted in an autosomal-recessive inheritance of the mutation following backcrosses from the original mixed background of C57BL/6129SvJ over >10 generations (614 wild-type animals, 303 heterozygous animals, 138 *shaky* animals). Sequencing of other candidate genes affected in startle disease excluded further pathogenic sequence variations, as only common single nucleotide variants were found (*Glrb*: exon 5, c.A555G, p.L163L, rs13477223; *SLC6A5*: exon 2, c.A109G, p.T37A, rs31048165).

To confirm that *shaky* results from defects in glycinergic transmission, heterozygous *shaky* mice were bred with heterozygous *spasmodic* or *oscillator* mice. *Spasmodic* carries a different missense mutation A52S in the GlyR α1 subunit that affects glycine efficacy but results in a mild phenotype with tremor episodes but normal life span for the animals (Buckwalter et al., 1994; Saul et al., 1994). Eight matings of *shaky and oscillator* heterozygotes generated 10 mice with severe motor deficits with onset of symptoms at P14 and a life span of 4 weeks (Fig. 1*D*). Genotyping of the pups exhibited heterozygosity of affected mice for the *oscillator* and *shaky* mutations (*Glra1^sh/ot^*), confirming the validity of this test for allelism. By contrast, heterozygous *Glra1^spd/sh^* mice survive like homozygous *Glra1^spd/spd^* mice with similar mild neuromotor deficits upon tactile or acoustic stimuli (Fig. 1*D*). Together, these data provided strong evidence that the pathophysiological mechanism in the spontaneous mouse model *shaky* is a missense mutation (Q177K) in *Glra1*.

### Motor deficits in homozygous *shaky* mice are improved by benzodiazepine treatment

Due to their impaired motor control, *shaky* mice performed very poorly on the rotarod test (*Glra1*^+/+^ time on rod, 288 ± 5 s; *Glra1^sh/sh^* time on rod, 15 ± 3 s; ***p = 6.935E-27, *t* test; Fig. 1*E*). Humans suffering from startle disease respond to treatment with benzodiazepines such as clonazepam, which improves symptoms by potentiating GABAergic transmission. Therefore, *shaky* mice were tested on the rotarod prior to (baseline) and after intraperitoneal injection of 0.5 mg/kg diazepam. After this treatment, *shaky* mice showed a significant improvement in their performance (after diazepam: *t* = 34 ± 7 s; *n* = 10; **p = 0.00102, *t* test) on the rod (Fig. 1*E*). Again, improvement of the symptoms following diazepam treatment provides further evidence for a glycinergic transmission defect in *shaky* mice.

### *Shaky* GlyRs have disrupted ligand binding, but the neuromotor phenotype does not result from disturbed receptor biogenesis

**Figure 2.**
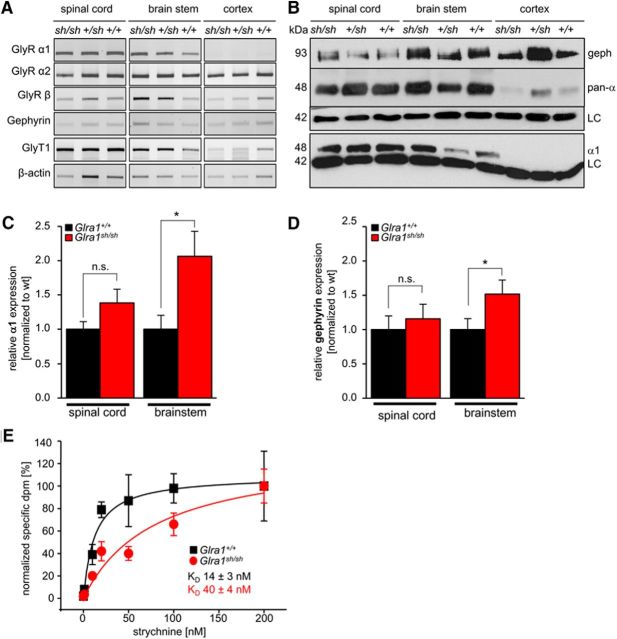
*Glra1^sh/sh^* mice display protein expression differences and reduced agonist binding. ***A***, Transcript analysis of GlyR α1, α2, and β subunits, the scaffold protein gephyrin, the glycine transporter 1 (GlyT1), and β-actin (housekeeping gene) in spinal cord, brainstem, and cortex from *Glra1*^+/+^ (marked +/+), *Glra1*^+/*sh*^ (+/*sh*), and *Glra1^sh/sh^* (*sh/sh*) mice. ***B***, GlyR protein expression profile in spinal cord, brainstem, and cortex at P21 in wild-type (+/+), heterozygous (+/*sh*), and homozygous animals (*sh/sh*). Gephyrin (geph) was detected at the appropriate molecular weight of 93 kDa; all GlyR α subunits were stained with a pan-α antibody (mAb4a, 48 kDa), GlyR α1 specifically with mAb2b (48 kDa); and β-actin was used as loading control (LC; 42 kDa). ***C***, ***D***, Quantification of GlyR α1 protein (***C***) and gephyrin (***D***) in spinal cord and brainstem normalized to β-actin in wild-type (*Glra1*^+/+^) and *shaky* (*Glra1^sh/sh^*; *n* = 6–10, *p < 0.05, n.s.). ***E***, Strychnine–glycine competition in spinal cord tissue isolated from wild-type (pooled, *n* = 3) and homozygous *shaky* mice (pooled, *n* = 4), three independent experiments were performed. Thirty millimolar glycine bound to membrane preparations of tissue were displaced by [^3^H]strychnine using increasing concentrations of the radioactive antagonist (0–200 nm). Note the increase in K_D_ in membrane preparations from *shaky* mice.

**Figure 3.**
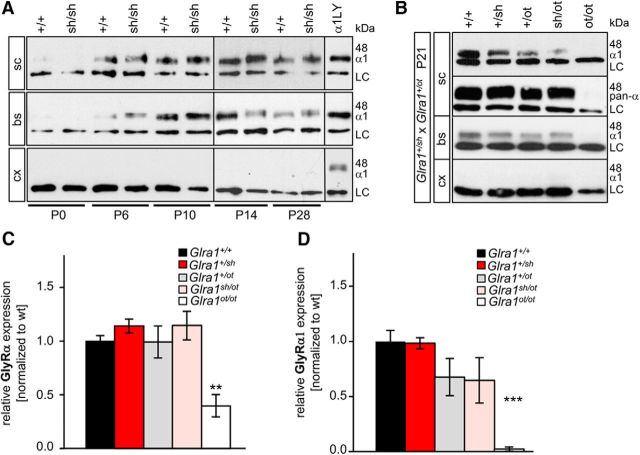
Developmental expression of the GlyR α1 subunit in *shaky* mice and after backcross into the mouse line *oscillator*. ***A***, Spinal cord (sc), brainstem (bs), and cortex (cx; negative control) were analyzed at developmental stages P0, P7, P14, and P28 until severe symptoms in homozygous *shaky* mice compared with wild-type mice were observed. The GlyR α1 subunit was stained with the monoclonal mAb2b antibody at 48 kDa, with β-actin was used as a loading control (LC; 42 kDa). α1LY represents a lysate control following transfection of GlyR α1 into HEK293 cells and was used as a positive control. ***B***, GlyR expression in P21 mouse tissue after backcross of *shaky* into the *oscillator* line. Sc, bs, and cx of mice carrying genotypes *Glra1*^+/+^, heterozygous *Glra1*^+/*sh*^, *Glra1*^+/*ot*^, *Glra1^sh/ot^*, and homozygous *Glra1^ot/ot^* were analyzed for the expression of GlyR α1 subunit (48 kDa). Note that homozygous *oscillator* mice *Glra1^ot/ot^* lack α1 and *Glra1^sh/ot^* as well as *Glra1*^+/*ot*^ reveal reduced GlyR α1 levels. Again, β-actin served as an LC (42 kDa). ***C***, ***D***, Quantification of GlyR α (pan-α antibody; ***C***) and GlyR α1 protein (***D***) from spinal cord preparations of *Glra1*^+/+^, heterozygous *Glra1*^+/*sh*^, *Glra1*^+/*ot*^, *Glra1^sh/ot^* animals, and homozygous *oscillator Glra1^ot/ot^* normalized to β-actin. The expression level of wild-type animals *Glra1*^+/+^ were set to 1 (100%), *n* = 4, *p < 0.05. n.s. = nonsignificant.

A first analysis of key proteins at the glycinergic synapse (GlyR α2, GlyR β, gephyrin, GlyT1) in wild-type (+/+), heterozygous (+/*sh*), and homozygous *shaky* (*sh/sh*) mice revealed no obvious differences at the mRNA level (Fig. 2*A*). At the protein level (Fig. 2*B–E*), the distinct expression of GlyR α subunits was detected in brainstem and spinal cord, but there was only faint expression in cortex (presumably, α2 or α3 subunits). Furthermore, specific α1 expression was enhanced in spinal cord of *Glra1^sh/sh^* (*p* = 0.09, nonsignificant, *t* test) and significantly increased in brainstem (*p = 0.019, *t* test) at P21 when *shaky* mice exhibit a severe neuromotor phenotype (Fig. 2*B*,*C*). No GlyR α1 expression was observed in cortex. Gephyrin was also increased concomitantly with GlyR α1 in the brainstem (*p = 0.045, *t* test) of affected animals (*Glra1^sh/sh^*; Fig. 2*B*,*D*). The broad expression of gephyrin in the cortex is consistent with a major role in GABA_A_ receptor clustering within this brain area (Tyagarajan and Fritschy, 2014). A developmental analysis of spinal cord, brainstem, and cortex from P0 to P28 indicated that GlyR α1 subunit expression in *Glra1^sh/sh^* was indistinguishable from that observed in *Glra1*^+/+^ mice and began at P6 (Fig. 3*A*). Furthermore, the subunit switch to increased GlyR α1 levels after birth was completed before the onset of symptoms at P14. Backcross into the *oscillator* line revealed a slight but nonsignificant decrease in GlyR α1 protein in heterozygous *Glra1*^+/*ot*^ and *Glra1^sh/ot^* animals, while the detection of all GlyR α variants in spinal cord tissue exhibited no differences among wild-type animals (*Glra1*^+/+^), heterozygous *shaky* (*Glra1*^+/*sh*^), *oscillator (Glra1*^+/*ot*^), and *Glra1^sh/ot^* animals (Fig. 3*B*,*C*). Due to the *Glra1* frameshift mutation in the *oscillator* line, *Glra1^ot/ot^* showed significantly reduced GlyR α1 levels (2.5 ± 1.3%) and 40 ± 17% GlyR expression levels corresponding to α2, α3, and β subunits (Fig. 3*C*,*D*; Kling et al., 1997).

**Figure 4.**
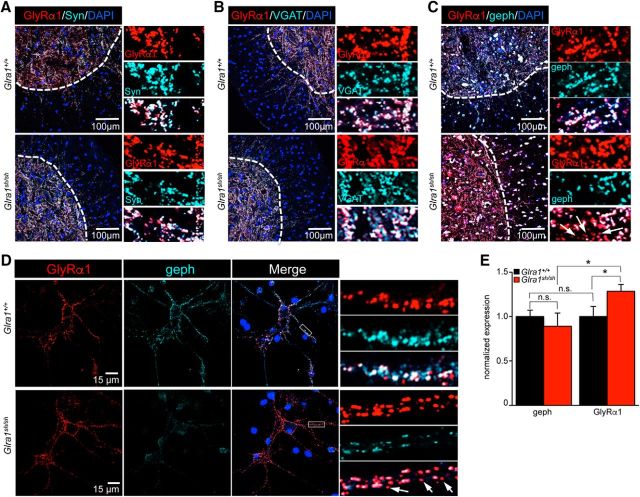
GlyR α1^Q177K^ results in a lack of synaptic integration. ***A–C***, Immunohistological stainings of spinal cord tissue from *Glra1*^+/+^ compared with homozygous *Glra1^sh/sh^* animals. Spinal cord slides (9 μm) were stained for the GlyR α1 subunit with the monoclonal antibody mAb2b together with presynaptic markers synapsin (syn; ***A***) vesicular transporter (VGAT; ***B***) and the postsynaptic marker gephyrin (geph; ***C***). Ventral or dorsal horns are marked by a white dotted line. DAPI was used to stain nuclei. Right panels represent enlarged images of each staining. Note that there is less colocalization of GlyR α1 and gephyrin in homozygous *shaky* mice (white arrows). ***D***, Spinal cord neuronal cultures from E13 embryos with genotypes *Glra1*^+/+^ or *Glra1^sh/sh^* were differentiated for 3 weeks in culture and stained for α1 (mAb2a) and gephyrin. An upregulation of GlyR α1 in *shaky* neurons was observed, but less colocalization in synaptic clusters with gephyrin (white arrows). Right panels represent enlarged dendrites of spinal cord neurons, which are marked by white boxes. ***E***, Quantification of synaptic clusters in neurons from *Glra1*^+/+^ and *Glra1^sh/sh^*, *n* = 11 from two independent experiments. The relative expression of gephyrin and GlyR α1 is shown (*p < 0.05, n.s.), comparison of expression levels between *Glra1*^+/+^ and *Glra1^sh/sh^* and within each group.

As a functional readout, we examined radioligand binding using spinal cord tissue from *Glra1^sh/sh^* mice compared with *Glra1*^+/+^ controls. When a saturating concentration of glycine (30 mm) was replaced by increasing concentrations of [^3^H]strychnine (Fig. 2*E*), a higher strychnine concentration was required to displace glycine from spinal cord membranes of *shaky* mice (*Glra1^sh/sh^*, 40 ± 4 nm; *Glra1*^+/+^, 14 ± 3 nm). Although GlyR α1 expression in *shaky* mice is increased, binding of the antagonist strychnine is diminished. To analyze synaptic localization, we performed immunostaining of spinal cord tissue and samples from spinal cord cultures from *Glra1*^+/+^ and *Glra1^sh/sh^* mice. In contrast to wild-type GlyR α1, a fraction of GlyR α1^Q177K^ was not colocalized with presynaptic markers synapsin and VGAT (Fig. 4*A*,*B*) or with the postsynaptic marker gephyrin (Fig. 4*C*). Quantification of synaptic GlyR α1 in neuronal cultures demonstrated a significant decrease in *Glra1^sh/sh^* compared with *Glra1*^+/+^ (gephyrin comparison between *Glra1*^+/+^ and *Glra1^sh/sh^*, *p* = 0.25, n.s.; GlyR α1 comparison between *Glra1*^+/+^ and *Glra1^sh/sh^*, *p = 0.018; gephyrin compared with GlyR α1 in *Glra1*^+/+^, *p* = 0.5; gephyrin compared with GlyR α1 in *Glra1^sh/sh^*, *p = 0.017, *t* test). An apparent increase in α1^Q177K^ expression observed in *shaky* neurons may result from an attempt to compensate for the lack of synaptic α1-containing GlyRs (Fig. 4*D*,*E*). Hence, GlyR α1^Q177K^ expression is enhanced in brainstem and spinal cord, but synaptic localization is decreased. At the functional level, GlyRs in *shaky* mice display a lower strychnine-binding affinity, suggesting an additive effect of sorting deficits and functional disruption.

### The α1^Q177K^ mutation impairs the functionality of GlyR channels *in vitro*

**Figure 5.**
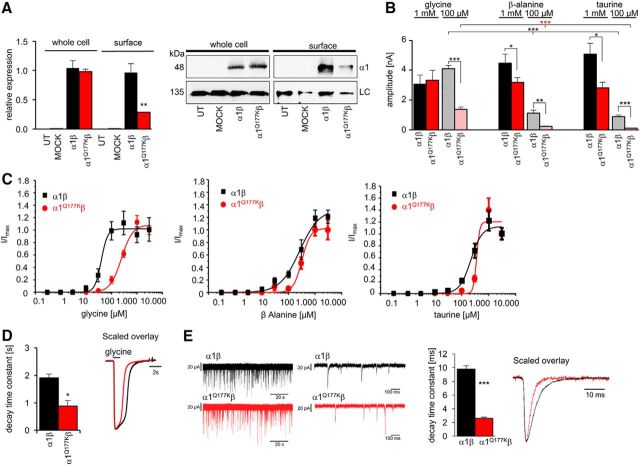
GlyR α1^Q177K^ leads to reduced agonist potency and faster decay times. ***A***, Expression of wild-type and mutant heteromeric receptor complexes following cotransfection with the GlyR β subunit in HEK293 cells. Biotinylation assays were used to discriminate between surface and whole-cell protein. Left, Quantification of wild-type and mutant GlyR α1 protein with or without the GlyR β-subunit from whole-cell and surface pools normalized to pan-cadherin; data were obtained from at least three independent sets of experiments (*n* = 3–6). Right, Western blot analysis of biotinylation assays. The monoclonal pan-α antibody was used for recognition of the GlyR α1 subunit (48 kDa), the membrane protein pan-cadherin served as a loading control (LC; 135 kDa). UT, Untransfected cells; MOCK, GFP-transfected cells. ***B***, Functional parameters from whole-cell recordings of transfected HEK293 cells for α1β and α1^Q177K^β heteromeric receptor configurations, with current amplitudes at 1 mm (α1β, *n* = 9; α1^Q177K^β, *n* = 16) and 100 μm glycine (α1β, *n* = 5; α1^Q177K^β, *n* = 5); 1 mm (α1β, *n* = 11; α1^Q177K^β, *n* = 9) and 100 μm β-alanine (α1β, *n* = 6; α1^Q177K^β, *n* = 6); and 1 mm (α1β, *n* = 6; α1^Q177K^β, *n* = 5) and 100 μm taurine (α1β, *n* = 5; α1^Q177K^β, *n* = 6). ***C***, Ligand-binding potencies (EC_50_) determined by current measurements of transfected cells expressing the heteromeric receptor configurations α1β and α1^Q177K^β according to the adult *in vivo* receptor configuration at seven different glycine, β-alanine, and taurine concentrations (0.3–3.000 μm, *n* = 5 for each receptor configuration and for the agonist used). The 1 mm concentration of the agonist glycine or the partial agonists β-alanine and taurine were used to determine *I*_max_ values. ***D***, Decay times for desensitization. The decay currents were calculated in the presence of agonist (1 s). Right, Representative scaled current traces with α1β (*n* = 5, black) and α1^Q177K^β (*n* = 6, red). ***E***, Faster decay of *shaky* channels expressed in artificial synapses. Spontaneous IPSCs of α1β (black) and α1^Q177K^β (red) shown at two different time scales, 20 s and 100 ms. Quantification of decay time constants of α1β (*n* = 10) and α1^Q177K^β (*n* = 9) in artificial synapses/ enlarged scaled view of α1β (black) and α1^Q177K^β (red). *p* < 0.05; **p < 0.01; ***p < 0.001.

Using overexpression of GlyRs in transfected HEK293 cells, quantification of whole-cell and plasma membrane protein of α1^Q177K^β GlyRs was compared with wild-type α1β receptors. This revealed a significant decrease of α1^Q177K^β surface expression (heteromeric α1^Q177K^β 33 ± 5% of wild-type α1β, **p = 0.003, *t* test; Fig. 5*A*). In whole-cell recordings from transfected cells, it became obvious that α1^Q177K^β cell surface GlyRs could form functional ion channels with no changes in maximal current amplitudes upon 1 mm glycine application (Fig. 5*B*) but with significantly lower current values at 100 μm glycine (α1β, 4.7 ± 0.3 nA; α1^Q177K^β, 1.13 ± 0.3 nA; ***p = 6.616E-06, *t* test; Fig. 5*B*). In contrast, on application of 1 mm β-alanine or taurine, α1^Q177K^β GlyRs exhibited significantly reduced current amplitudes (β-alanine, 3.2 ± 0.3 nA, *n* = 11; compared with α1β, 4.5 ± 0.6 nA, *n* = 9; *p = 0.033, *t* test; taurine, 2.8 ± 0.4 nA, *n* = 6; compared with α1β, 5.1 ± 0.7 nA, *n* = 5, *p = 0.014, *t* test). A concentration of 100 μm for both partial agonists generated again significantly decreased agonist-induced currents compared with wild-type receptors (β-alanine α1^Q177K^β, 0.23 ± 0.02 nA, *n* = 6; compared with α1β, 1.1 ± 0.2 nA, *n* = 6, **p = 0.0014, *t* test; taurine, α1^Q177K^β 0.1 ± 0.02 nA, *n* = 6; compared with α1β, 0.9 ± 0.09 nA, *n* = 5, ***p = 6.72E-06, *t* test; Fig. 5*B*).

Hence, the EC_50_ value for mutated α1^Q177K^β channels activated by glycine increased by a factor of 6 (α1β EC_50_ = 40 ± 6 μm; α1^Q177K^β EC_50_ = 241 ± 29 μm; Fig. 5*C*). The potencies of the partial agonists β-alanine (α1^Q177K^β EC_50_ = 318 ± 22 μm; α1β EC_50_ = 260 ± 50 μm) and taurine (α1^Q177K^β EC_50_ = 389 ± 51 μm; α1β EC_50_ = 212 ± 34 μm) were almost unaffected with a slight decrease of 1.2- and 1.8-fold, respectively (Fig. 5*C*).

Desensitization decay time constants were significantly decreased for mutant receptors, arguing for faster ion channel closure of α1^Q177K^β heteromers compared with wild-type channels (α1β τ = 1.9 ± 0.14 s; α1^Q177K^β τ = 0.88 ± 0.19 s; *p = 0.049, *t* test; Fig. 5*D*). Last, artificial synapses were used to analyze the α1^Q177K^ mutation in the context of spontaneous glycine release from neighboring spinal cord interneurons. Spontaneous IPSPs (IPSCs) of α1^Q177K^β compared with α1β exhibited again a significant decrease in the decay time constant (***p = 5.2066E-5, *t* test; Fig. 5*E*).

### The mutation Q177K disrupts GlyR function by faster ion channel closure

**Figure 6.**
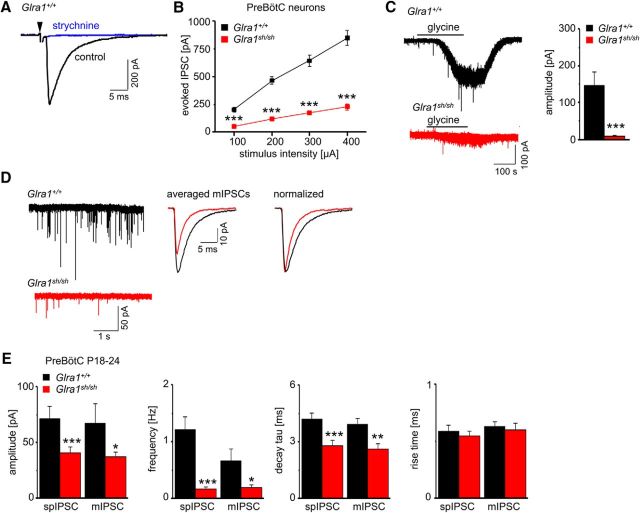
*Shaky* mice have impaired glycinergic synaptic transmission in PreBötC neurons. All recordings were made in whole-cell voltage-clamp mode in the presence of the ionotropic glutamate receptor antagonist KA (2 mm) and the GABA_A_ receptor antagonist bicuculline (10 μm). ***A***, Traces from a wild-type PreBötC neuron illustrate the evoked IPSC (at stimulation intensity of 200 μA for 0.1 ms) and sensitivity to strychnine (10 μm). ***B***, Input–output curves of evoked IPSCs in *Glra1*^+/+^ (*n* = 24 from 12 mice) and *Glra1^sh/sh^* (*n* = 25 from 12 mice) mice; comparison of *Glra1*^+/+^ and *Glra1^sh/sh^* at 100 μA: ***p = 3.198E-09; at 200 μA, ***p = 1.539E-12; at 300 μA, ***p = 3.20E-12; at 400 μA, ***p = 3.32E-12, *t* test). ***C***, Traces from wild-type and *Glra1^sh/sh^* neurons show postsynaptic current responses to glycine application (50 μm), recorded in TTX (1 μm). Note the increase in baseline noise with glycine in *Glra1^sh/sh^* neuron, despite no shift in the holding current. Diagram on the right summarizes glycine-induced currents (*Glra1*^+/+^, *n* = 7 from 3 mice; *Glra1^sh/sh^*, *n* = 9 from 4 mice). ***D***, Traces of spontaneously occurring mIPSCs, recorded in TTX (1 μm) from both genotypes, illustrate the reduced action potential-independent synaptic activity in *Glra1^sh/sh^* mice. Averaged mIPSCs from the two neurons were superimposed and normalized, revealing the strong decrease in amplitude and acceleration in decay for *Glra1^sh/sh^* mIPSCs. ***E***, Quantitative analysis of spIPSCs (without TTX) and mIPSCs in PreBötC neurons demonstrates significant changes in properties and kinetics of spontaneously occurring events (spIPSCs: *Glra1*^+/+^, *n* = 17 from 10 mice; *Glra1^sh/sh^*, *n* = 21 from 12 mice; mIPSCs: *Glra1*^+/+^, *n* = 6 from 3 mice; *Glra1^sh/sh^*, *n* = 6 from 4 mice). *p < 0.05; **p < 0.01; ***p < 0.001.

To investigate glycinergic synaptic signaling in intact synapses *in situ*, we prepared brainstem slices from wild-type and *shaky* mice and performed whole-cell recordings from neurons of the PreBötC, a nucleus rich in GlyR α1 expression and important for respiration. When strychnine-sensitive glycinergic IPSCs (Fig. 6*A*) were evoked at different stimulus intensities in PreBötC neurons at P18 to P24, a dramatically flattened input–output relationship in the mutant preparation was obtained (***p < 0.001 for all stimulus intensities; for values see the legend of Fig. 6*B*). Bath-applied glycine (50 μm) caused a significant smaller baseline shift in *Glra1^sh/sh^* neurons compared with control, again suggesting a deficit in postsynaptic GlyR function (***p = 0.0004, *t* test; Fig. 6*C*). mIPSC recordings (in 1 μm TTX) from PreBötC neurons to study individual glycinergic synapses revealed significantly lower frequency, smaller amplitudes, and accelerated decay in *Glra1^sh/sh^* neurons (mIPSCs: *Glra1*^+/+^ compared with *Glra1^sh/sh^*, amplitude *p = 0.032; frequency, *p = 0.031; decay, **p = 0.0017; rise time, *p* = 0.423, nonsignificant). These effects were independent from the block of spontaneous spiking by TTX (spIPSCs; *Glra1*^+/+^ compared with *Glra1^sh/sh^*: amplitude, ***p = 4.27E-06; frequency, ***p = 2.55E-07; decay, ***p = 2.58E-06; rise time, *p* = 0.211, nonsignificant; Fig. 6*D*,*E*).

**Figure 7.**
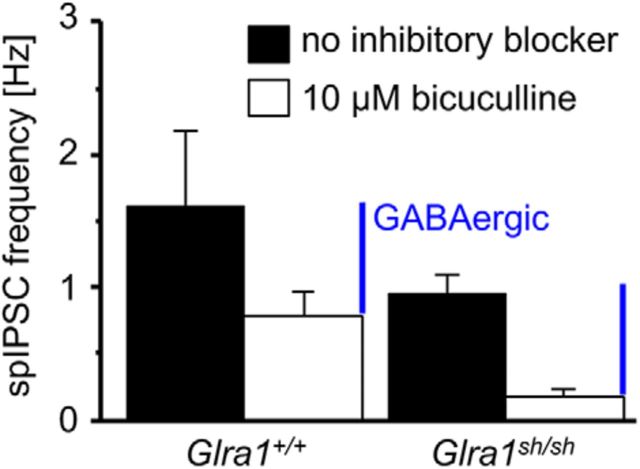
The *shaky* mutation does not affect GABAergic synaptic transmission in the PreBötC neurons. All recordings were made in the presence of KA (2 mm). The frequency of GABAergic spIPSCs was calculated by comparing and subtracting the spIPSCs before and after bicuculline (10 μm) application in the same neuron. PreBötC neurons from *Glra1^sh/sh^* mice have GABAergic spIPSCs at a frequency of 0.76 ± 0.18 Hz (*n* = 12 from 9 mice), which is not different from that observed in *Glra1*^+/+^ neurons (0.78 ± 0.19 Hz, *n* = 4 from 4 mice).

**Figure 8.**
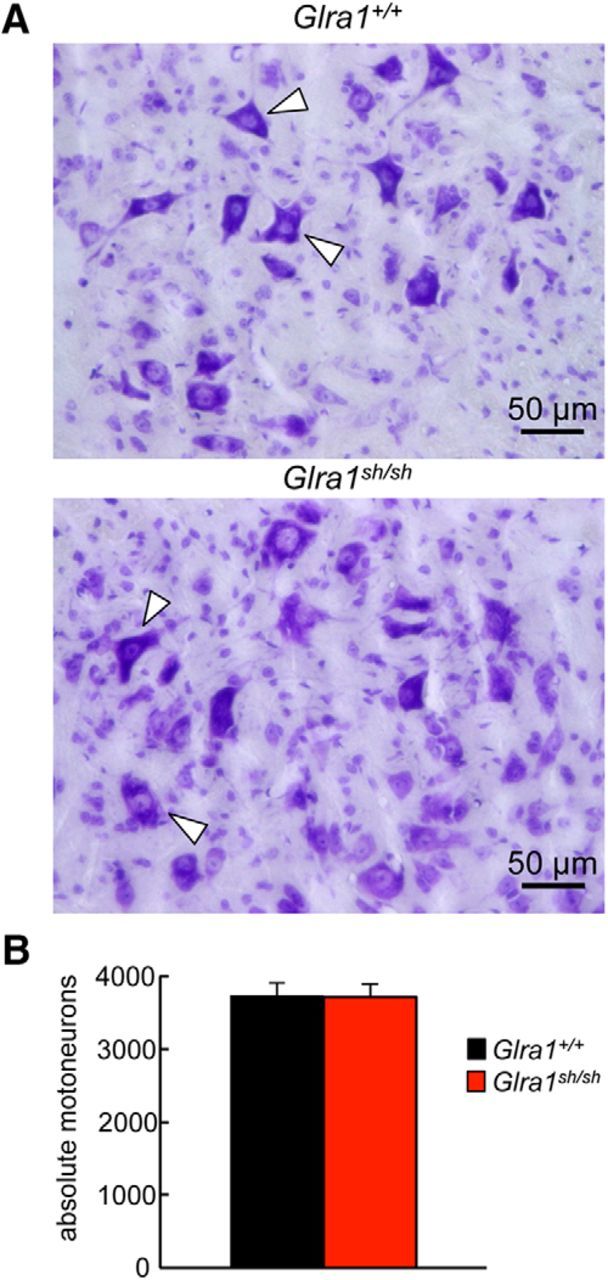
*Glra1*^+/+^ and *Glra1^sh/sh^* do not differ in motoneuron numbers. Lumbar spinal cord of *Glra1*^+/+^ and *Glra1*^sh/sh^ at P28 was cut in 12.5 μm slices, and the numbers of motoneurons were counted. Spinal cord slices were stained with cresyl violet, and motoneurons were determined due to their large cell body (white arrowheads). ***A***, Representative pictures from *Glra1*^+/+^ and *Glra*1^sh/sh^ slices with large cell bodies of motoneurons. ***B***, Quantitative analysis of motoneuron numbers. The quantification was made using six to seven animals of each genotype (*Glra1*^+/+^, *n* = 6; *Glra1*^sh/sh^, *n* = 7). Error bars represent SDs.

Many PreBötC neurons receive mixed GABAergic/glycinergic inhibitory synaptic input with a corelease of both neurotransmitters from the same synaptic terminal (Rosenmund and Stevens, 1996). GABAergic spIPSCs, however, did not differ in frequency between the genotypes (*p* = 0.067, nonsignificant; Fig. 7), arguing against a generalized reduction in inhibitory synaptic terminal number or release probability as the main factor behind defects in glycinergic inhibition. To further exclude an overall decrease in motoneuron number as a modifier of the underlying pathology in *shaky* mice, the number of motoneurons was counted in lumbar spinal cord. No differences between *Glra1*^+/+^ and homozygous *Glra1^sh/sh^* animals were observed in six to seven animals analyzed for each genotype (*p* = 0.44, nonsignificant, *t* test; Fig. 8*A*, *B*). To conclude, the dramatically decreased amplitudes in glycine-evoked currents, mIPSCs, and spIPSCs explain the severity of the observed *shaky* phenotype. Similar to recordings from overexpressed cells as well as artificial synapses, decay times were accelerated in brainstem slice recordings, suggesting a function of the GlyR β8–β9 loop in the glycinergic signal transduction pathway.

### Q177K disrupts hydrogen bonding with residues in the ligand-binding pocket

**Figure 9.**
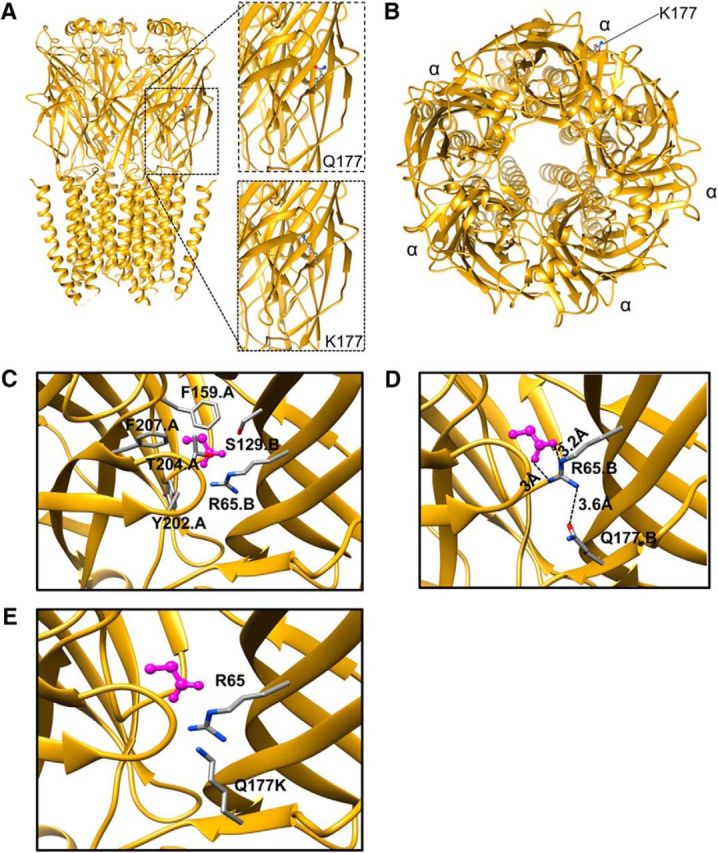
GlyR α1^Q177K^ disrupts hydrogen bonding with the key ligand binding residue R65. ***A***, Cryo-EM model 3JAE with the wild-type GlyR α1 (α1^Q177^, top right) and the modeled *shaky* mutation (α1^K177^, bottom right). ***B***, Top view of the homo-pentameric arrangement and position of Q177K. ***C***, Glycine-binding pocket in the GlyR structure, where the glycine ligand is shown in magenta, and surrounding residues are labeled appropriately. ***D***, Position of Q177 based on the cryo-EM model shown relative to the new position of R65 derived upon flexible fitting into the cryo-EM density using Flex-EM (Topf et al., 2008; Joseph et al., 2016). Interactions are indicated for Q177 with R65 from the glycine-binding pocket. ***E***, The modeled Q177K substitution.

The recently uncovered cryo-EM structure of zebrafish GlyR α1 (PDB ID, 3JAE) provided evidence that the β8–β9 loop harboring the Q177K mutation is involved in ion channel opening/closing processes (Du et al., 2015). Glycine was modeled into the GlyR structure based on the position of glutamate in the binding pocket of GluCl (Hibbs and Gouaux, 2011) by superposition of GluCl (PDB ID, 3RHW) onto the GlyR structure. The Q177K substitution was inserted into the glycine-bound open-channel state as part of β-strand 9 (Fig. 9*A–C*). The glycine-binding pocket comprises residues R65 and S129 from one subunit (*B*) and F159, Y202, T204, and F207 from the adjacent subunit (*A*; Fig. 9*C*). R65 is crucial for ligand binding as it interacts with the α-carboxyl and α-amino groups of glycine (Grudzinska et al., 2005). The guanidinium group of R65 has an electrostatic interaction with the glycine carboxylate group (in the pocket), but also forms an additional H-bond with the Q177 side chain (Fig. 9*D*). The Q177K substitution is predicted to not only abolish the formation of the H-bond between Q177 and R65 but is also likely to alter the position of R65 side-chain due to the additional positive charge contributed by the lysine side-chain. This in turn could lead to destabilization of the glycine-binding pocket (Fig. 9*E*). In summary, disrupted hydrogen bonding affecting key ligand-binding residues is likely to underlie the functional impairment shown by biochemical and physiological approaches and explains the severity of the *shaky* phenotype.

## Discussion

Our results comprehensively illustrate the importance of the β8–β9 loop structure within the extracellular domain for GlyR ligand binding and the subsequent conformational changes enabling signal transduction and synaptic clustering. The current understanding of GlyR ion channel function suggests the coupling of movements within the ECD (loop C, β1–β2, β6–β7) upon ligand binding proceeding to elements of the ECD–transmembrane domain interface (β10–pre-M1, the M2–M3 loop). These conformational rearrangements result in tilting of transmembrane domains, enabling ion channel opening and closing (Hassaine et al., 2014; Du et al., 2015; Gielen et al., 2015; Huang et al., 2015). The role of the β8–β9 loop, a flexible region localized at the complementary side of adjacent subunits underneath the ligand-binding pocket, has remained elusive due to the overall low electron density observed for this unstructured region of the ligand-binding site in earlier studies (Brejc et al., 2001; Hansen et al., 2005). Here, using the *shaky* mutant mouse that harbors a β8–β9 loop alteration, we found *in vivo* evidence that this extracellular loop is involved in stabilizing the GlyR ligand-binding site and ion channel gating. The pathogenic mechanism in this mouse mutant delineates a combined pattern of biogenesis defects resulting in defective synaptic integration, together with lower opening frequency, smaller amplitudes, and accelerated decay rates for remaining GlyRs, functional disturbances that are incompatible with survival.

In humans and rodents, mutations in GlyR subunit genes are associated with startle disease, characterized by exaggerated responses to acoustic stimuli and uncontrolled falling (Bode and Lynch, 2014) with an increased risk of cognitive deficits, such as learning difficulties or delay in speech acquisition (Thomas et al., 2013). The phenotype of the *shaky* mouse is in line with that of human patients, as well as strychnine poisoning (Buckwalter et al., 1994) and with other glycinergic mouse models (e.g., *spastic*, *oscillator*, and *Nmf11*; Traka et al., 2006; Schaefer et al., 2012). Our *in vitro* analysis revealed differences in surface expression between heteromeric wild-type α1β and mutant α1^Q177K^β GlyRs. Mutant α1^Q177K^β GlyRs were able to form functional ion channels with increased EC_50_ values. However, the desensitization time constant decreased for α1^Q177K^β GlyRs, indicating faster ion channel closure. The observed changes *in vitro* provided some explanations for the glycinergic defect present *in vivo*; however, the lethality of homozygous *shaky* mice presented a conundrum. A fivefold to sixfold reduction of GlyR agonist potency has also been documented in s*pasmodic* mice carrying an A52S substitution in the β1–β2 loop of the GlyR α1 subunit ECD. In contrast to *shaky* mice, homozygous *spasmodic* mice display a normal life span and a mild neuromotor phenotype, arguing that the shift in agonist potency alone cannot explain lethality (Graham et al., 2011). Moreover, *in vivo* expression levels of α1^Q177K^ were increased in spinal cord and brainstem, although synaptic integration was significantly diminished. Synaptic integration of GlyRs is enabled by the scaffolding protein gephyrin via high-affinity binding to the intracellular M3–M4 domain of the GlyR β subunit (Triller and Choquet, 2005; Dumoulin et al., 2010). A consequence of lower synaptic integration is less synaptic strength, which is normally regulated by lateral diffusion of synaptic and extrasynaptic receptors in and out of synapses (Triller and Choquet, 2005). The lower number of synaptic complexes argues that the extracellular binding site mutation is able to transduce conformational changes to the M3–M4 loop domain, thereby disrupting gephyrin binding and, thus, synaptic anchoring of α1^Q177K^β GlyRs. In turn, these data suggest that glycine sensitivity or gating properties of the receptor can be altered by the clustering status of the GlyR at the synapse. Another option that may explain the reduced synaptic integration is an enhanced turnover of the synaptic receptor pool via endocytosis as a neuronal adaptation to the impaired functionality of α1^Q177K^β GlyRs *in vivo*. We therefore conclude that the enhanced expression of α1^Q177K^ represents an unsuccessful attempt at neuronal compensation.

In addition to the postsynaptic effects of the *shaky* mutation, there may also be presynaptic consequences for functionally impaired homomeric α1^Q177K^ GlyRs. A potential role for presynaptic homomeric GlyRs in hyperekplexia has recently been demonstrated (Xiong et al., 2014). Presynaptic GlyR α1 subunit homomers have been described in calyceal synapses in the medial nucleus of the trapezoid body, in spinal cord and the ventral tegmental area (Turecek and Trussell, 2001; Jeong et al., 2003). Activation of these presynaptic GlyRs by glycine spillover triggers weakly depolarizing Cl^−^ currents. The generated depolarization leads to enhanced transmitter release by Ca^2+^ channel activation and increased Ca^2+^ concentrations in the nerve terminal (Turecek and Trussell, 2001). The increase in α1^Q177K^ expression *in vivo* may result in an enhanced expression of presynaptic homomeric GlyRs generating impaired presynaptic GlyR activity and thus, diminished glycine release in the brainstem and spinal cord of *shaky* mice. Consequently, disrupted presynaptic GlyR function would be consistent with the significantly reduced IPSC frequencies we observed in brainstem slice recordings.

Functional analysis in brainstem nuclei that are rich in glycinergic synapses revealed largely reduced current amplitudes and significantly lower frequencies of spontaneous and miniature IPSCs in *shaky* mice, which are likely to be consequences of the low numbers of postsynaptic functional receptors and enhanced expression of functionally impaired presynaptic homomeric GlyRs. As the reduced ligand potency of *shaky* GlyRs is still within the range of glycine concentrations that can be achieved during synaptic activation, the observed functional deficits must result from disturbed translation of ligand binding into ion channel opening (gating). Impaired gating was further confirmed by reduced efficacies of the partial agonists β-alanine and taurine. Moreover, residue Q177 normally undergoes H-bond formation with the ligand-binding residue R65, which is disrupted by the positively charged lysine. The positional change of the R65 side-chain due to the introduction of additional positive charge from K177 would in turn destabilize the glycine-binding pocket, which is in agreement with the observed decrease in glycine potency. The importance of the β8–β9 loop contribution to a hydrogen bond network in bound and unbound receptor states has been shown previously for γ2 GABA_A_ and 5HT_3A_ as well as nAChR subunits (Nys et al., 2013).

The observed decrease in the decay time constant for the α1^Q177K^ mutation, resulting in faster ion channel closure is similar to *spasmodic* mice (Graham et al., 2006), arguing for a significant impact of ion channel decay mechanisms on the startle phenotype and in the case of *shaky* a contribution to the lethality in this mouse model. The functional analogy between *spasmodic* and *shaky* mice suggests a defect in the same signal transduction pathway determined by coupling of ECD movements following ligand binding to finally ion channel opening and closing (Du et al., 2015). Functional synaptic α1^Q177K^ GlyRs close much faster than wild-type channels, implying a fast unbinding process of the agonist. Since binding of glycine was only marginally affected in spinal cord tissue, the functional defects of α1^Q177K^ must result from downstream processes (e.g., fast transduction of ligand-bound receptor into the closed conformation). These data are in line with the recently proposed model of signal transduction for GlyRs (Du et al., 2015; Morales-Perez et al., 2016; Nys et al., 2016). Thus, the integrity of the β8–β9 loop is a prerequisite for conformational rearrangements and is crucial during gating processes of the GlyR channel, which opens a novel window for therapeutics, resulting in prolonged open times. Accordingly, both the reduced synaptic integration and faster ion channel closure observed for GlyRs in *shaky* mice may also represent novel pathogenic mechanisms for human startle disease mutations. Together, our data reveal that the β8–β9 loop in the GlyR α1 ECD is a key regulator of glycinergic signaling. Furthermore, *shaky* represents the first *in vivo* mouse model demonstrating the incompatibility of a disrupted β8–β9 loop with life.
